# Heavily Ossified Soft Tissue Chondroma of Plantar Foot and the Significance of Radiological Imaging: A Case Report

**DOI:** 10.7759/cureus.37914

**Published:** 2023-04-21

**Authors:** Rimsha R Vohra, Muhammad Haseeb, Mohammad Owais, Syed Faqeer Hussain Bokhari, Khizer Yaseen

**Affiliations:** 1 Internal Medicine, Dow University of Health Sciences, Karachi, PAK; 2 Internal Medicine, Jinnah Hospital Lahore, Lahore, PAK; 3 Internal Medicine, Bahria International Hospital, Lahore, PAK; 4 Medicine, Mayo Hospital, Lahore, PAK; 5 Medicine and Surgery, Mayo Hospital, Lahore, PAK; 6 Internal Medicine, Hamdard College of Medicine and Dentistry, Karachi, PAK

**Keywords:** chondrocytes, enchondral ossification, calcified plantar fibromatosis, benign cartilaginous tumor, plantar foot chondroma, soft tissue chondroma

## Abstract

Soft tissue chondroma is a relatively rare, slowly growing, benign cartilaginous tumor. This solitary mass can imitate chondrosarcomas in radiologic and histological characteristics. The diagnosis is hard to establish on clinical presentation and relies on careful radiological examination. The lesion is equally prevalent in both genders and primarily affects people in their forties and sixties. They may occur in any part of the body; however, they are most commonly observed in hand and foot. We report the case of a 61-year-old female who presented with heavily ossified soft tissue chondroma within the plantar fascia of her left foot. A conclusive diagnosis was established via histopathological examination. The chondroma was marginally excised, and the postoperative period was uneventful.

## Introduction

Chondroma is a benign cartilaginous tumor that develops from bone [1]. It has an incidence of 1.5% of all neoplasms [2]. Soft tissue chondromas (STC) are exceedingly rare benign solitary cartilaginous tumors that develop in soft tissues [3]. These tumors are rare in children and mostly seen in adults, especially between the fourth and sixth decade, without any sexual preference [3-5]. They may develop from various kinds of tissues, with exception of bone and cartilage. Hands and feet are the most commonly affected regions [4]. Clinical presentation is variable depending on the site of occurrence, such as pain while walking and difficulty with footwear [6]. Ultrasonography and MRI are used to establish an initial diagnosis, whereas histopathological examination leads to a conclusive diagnosis [1,7,8]. Surgical excision of the lesion is required to relieve symptoms [9]. We hope that this case report will add to the existing literature and help devise a comprehensive diagnostic and therapeutic plan for soft tissue chondromas.

## Case presentation

**Figure 1:**
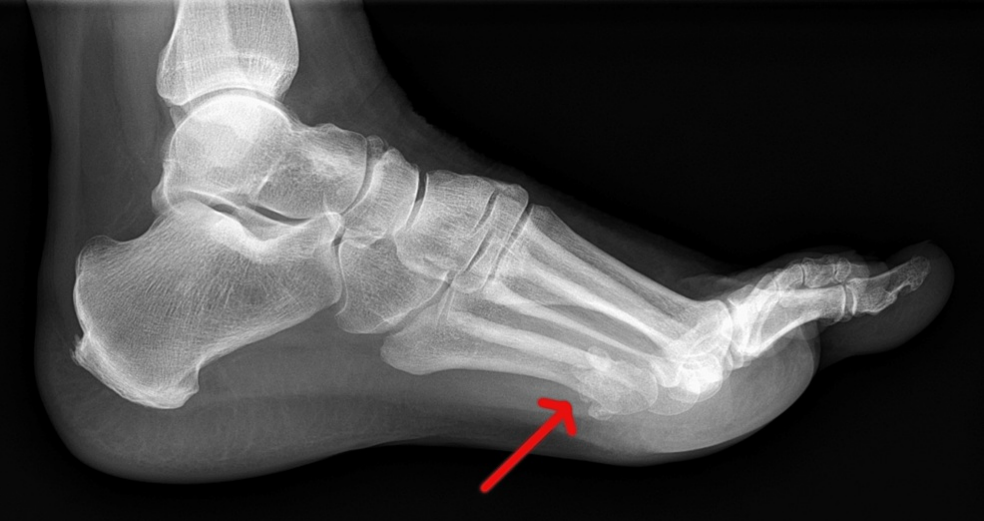
Lateral X-ray of the left foot The image shows well-corticated ossicles (arrow) in relation to the mid aspect of the 3rd and 4th metatarsal.

**Figure 2:**
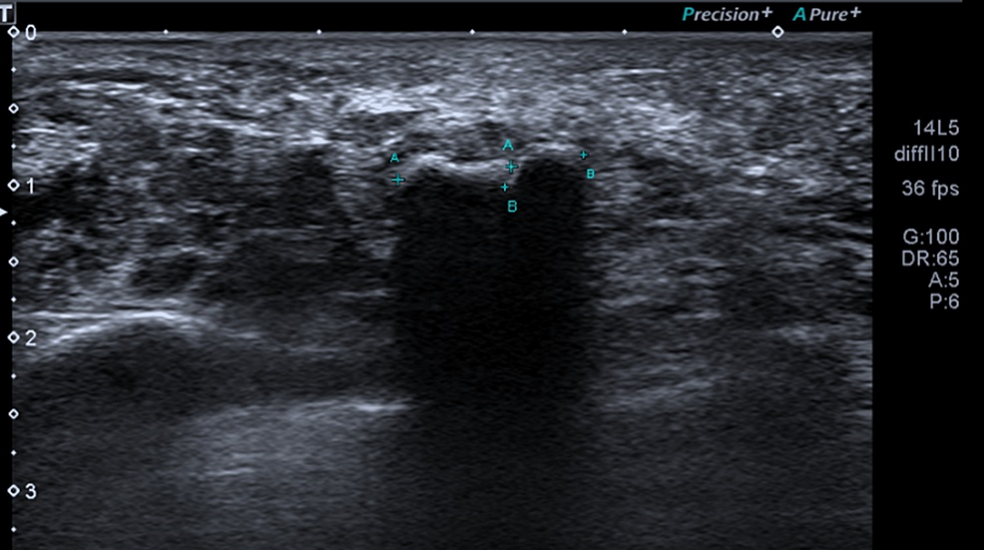
Ultrasound of the left foot Ultrasound of the left foot is suggestive of calcified plantar fibromatosis. (A-A and B-B)

A 61-year-old female presented in outpatient department with a long-standing symptomatic mass on the plantar aspect of her left foot in the area of 3rd and 4th metatarsal. The patient noticed this lesion two years prior to the examination due to persistent pain and was treated conservatively with changing shoe gear, padding and orthosis. There was associated tenderness in the involved region, and pain (diffuse) was more severe when bearing weight. Vitals of patient were stable and initial blood workup was normal. Multiple well corticated ossicles in relation to the mid aspect of the 3rd and 4th metatarsal on the plantar side were observed on radiological examination (Figure 1). Ultrasonography revealed three foci of calcification measuring 9x7 mm, 8 mm and 5 mm which appeared to be in relation to the plantar fascia, approximately 15 mm from the 1st metatarsophalangeal joint. It was suggestive of calcified plantar fibromatosis (Figure 2). 

Corticosteroid injections were discussed as a treatment option, but the patient refused. Surgical excision of the mass was carried out. Tibial nerve block was done using naropin, and further regional block was done in a V fashion proximal to incision using dexamethasone and naropin combination. Lazy S incision was made overlying the palpable mass. The incision was deepened through the subcutaneous tissue. Dissection carried down to the level of plantar fascia where upon palpation, it was noted that the lesion was in between the plantar fascia and the flexor digitorum brevis muscle belly. The bony lesion measuring 23x13x9 mm was dissected and passed on from the operative field for histopathology. 

On histopathological examination, the excision showed a benign multinodular osteocartilagenous lesion. The nodules were formed by a peripheral rim of lobulated hyaline cartilage merging with trabeculae of woven and lamellar bone through enchondral ossification. Within the cartilaginous matrix, a mildly cellular population of bland chondrocytes occurring in nests and situated within lacunar spaces was observed (Figure 4). Despite the suspicion of calcified plantar fibromatosis on ultrasound, there was no spindle cell proliferation in this case to suggest fibromatosis. The lesion appeared to be marginally excised. Hence, it was confirmed that the lesion was a heavily ossified soft tissue chondroma.

**Figure 3:**
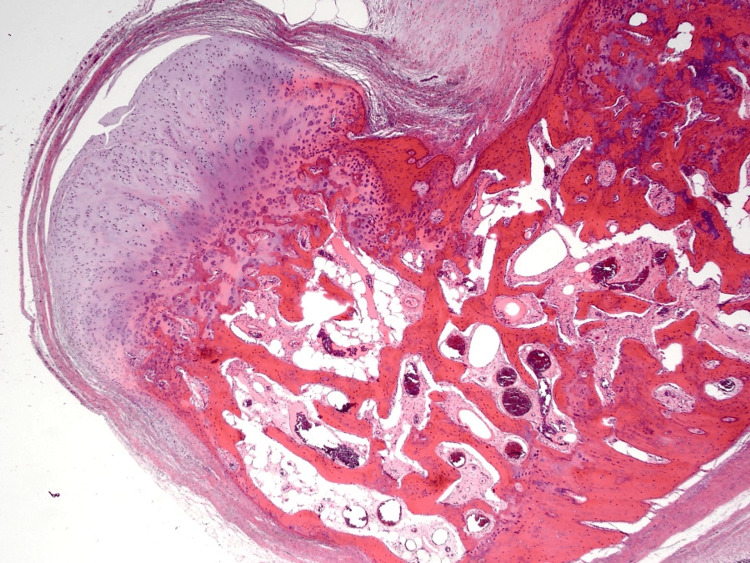
H&E staining of the lesion H&E: Hematoxylin and Eosin The image shows the peripheral rim of lobulated hyaline cartilage merging with trabeculae of woven and lamellar bone through enchondral ossification. Within the cartilaginous matrix, a mildly cellular population of bland chondrocytes occurring in nests and situated within lacunar spaces can be observed.

## Discussion

Soft tissue chondroma (STC or extra skeletal chondroma) is a soft-tissue tumour that is extremely rare [1,10,11]. Baumuller was the first to report it in 1883, and roughly 200 instances have been documented since then [10]. These tumours can affect people of any age, although they are more frequent in those between the ages of 30 and 60 years old [1,10,12]. The hands and feet are the most typical areas of occurrence [10]. Soft tissue chondroma has an unknown origin. According to the research on tongue chondromas, these lesions either form from leftover embryonal tissue in an area of pre-existing fetal cartilage or pluripotent mesenchymal cells undergo metaplasia, developing into cartilage in response to some irritating stimuli [13]. However, the precise causal link is unknown.

Clinically, the tumour appears as a single, slowly growing solid or rubbery well-defined mass [14]. It develops slowly in soft tissue without involving the bones and is typically asymptomatic and unnoticed until a tumor forms. Because these tumors are initially asymptomatic until a mass develops, the clinical history is frequently characterised by a painless, slowly expanding soft tissue mass [3]. When the lesion is on the plantar aspect of the foot, it might be uncomfortable [15,16]. The tumor is a painless, hard, well-delimited nodular mass with a fluctuating size when palpated. Glomus tumour, eccrine poroma, epidermal cyst, osteoma cutis, and calcinosis cutis should all be considered in the clinical differential diagnosis [17].

Radiological and histological findings are of paramount importance in establishing diagnosis of STC. A well-demarcated extraosseous soft tissue mass can be seen on radiographs [14]. Calcifications are frequently visible on simple X-rays, as in this case. Because of its rarity and lack of typical characteristics other than calcification, preoperative identification of soft tissue chondroma is difficult [18]. Lobules of mature (chondrocytes) or immature (chondroblasts) hyaline cartilage with variable degrees of cellularity can be detected on histological examination [19]. Hyaline, fibrous, or fibrohyaline matrixes can be used [14]. Immunohistochemistry is beneficial in the diagnosis of STC in addition to histopathologic evaluation, because tumor cells are positive for S-100 protein and vimentin but negative for epithelium and myoepithelial cell markers [20]. 

Despite STC being a benign tumour, thorough excision should be performed to relieve any symptoms such as pain or struggle with footwear [10]. It is recommended to entirely remove the tumor, including the capsular structure and adhesion sites, to avoid local recurrence.

## Conclusions

Soft tissue chondroma is a rare benign tumor that mostly affects the extremities, such as the hands and feet. Clinically, soft tissue chondroma of the foot presents as a mildly painful cutaneous lump. Therefore, In individuals with slow-growing and mildly painful cutaneous nodules, STC should be included in differential diagnosis. Preoperative diagnosis is difficult to establish; however, calcifications are clearly visible on radiological examination, which may indicate STC. Surgical excision of the mass is the treatment of choice to relieve symptoms. Histological examination of the lesion aids in forming a conclusive diagnosis. Postoperative period is normal and recurrence is rare.
